# Evolutionary developmental genetics of fruit morphological variation within the Solanaceae

**DOI:** 10.3389/fpls.2015.00248

**Published:** 2015-04-13

**Authors:** Li Wang, Jing Li, Jing Zhao, Chaoying He

**Affiliations:** ^1^State Key Laboratory of Systematic and Evolutionary Botany, Institute of Botany – Chinese Academy of Sciences, BeijingChina; ^2^Graduate University of Chinese Academy of Sciences, BeijingChina

**Keywords:** domestication, evolutionary developmental genetics, fruit, gene expression, morphological novelty, natural variation, Solanaceae

## Abstract

Morphological variations of fruits such as shape and size, and color are a result of adaptive evolution. The evolution of morphological novelties is particularly intriguing. An understanding of these evolutionary processes calls for the elucidation of the developmental and genetic mechanisms that result in particular fruit morphological characteristics, which determine seed dispersal. The genetic and developmental basis for fruit morphological variation was established at a microevolutionary time scale. Here, we summarize the progress on the evolutionary developmental genetics of fruit size, shape and color in the Solanaceae. Studies suggest that the recruitment of a pre-existing gene and subsequent modification of its interaction and regulatory networks are frequently involved in the evolution of morphological diversity. The basic mechanisms underlying changes in plant morphology are alterations in gene expression and/or gene function. We also deliberate on the future direction in evolutionary developmental genetics of fruit morphological variation such as fruit type. These studies will provide insights into plant developmental processes and will help to improve the productivity and fruit quality of crops.

## Introduction

Diversification of plant morphology occurred during evolution as a result of plant adaptation to changes in the environment. The origin of the fruit is an evolutionary adaptation that facilitates survival and distribution of progeny. For example, fruits protect the developing seeds from adverse environments and/or foraging by animals during premature stages, thus enhancing the survival rate. However, fruits that contain nutrients and minerals can become favorite foods for animals and humans as part of a balanced diet. The energetic cost of producing fruits are paid for through subsequent seed dispersal, e.g., birds, mammals, and humans disperse seeds to different habitats where they can propagate. In some cases, the origin of morphological novelties or particular structures associated with fruits play an essential role in seed dispersal by wind, water and animals. Diverse colors and flavors of mature fruits attract animals that eat the fruit and aid seed dispersal. Thus, the morphological variations of fruits have diversified considerably. Furthermore, humans have domesticated a wide range of plants as fruit crops with different sizes, shapes, colors, flavors, and textures.

**FIGURE 1:**
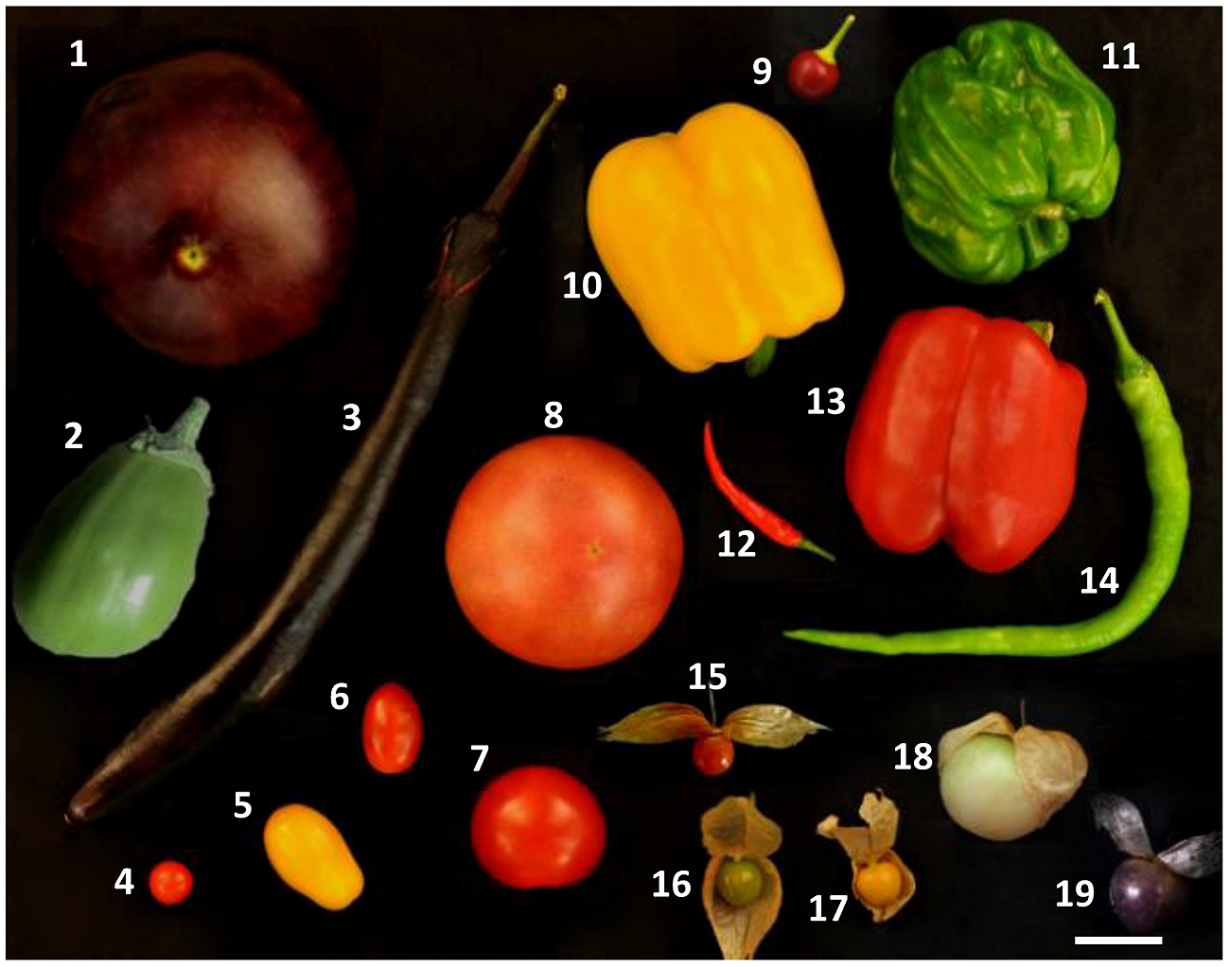
**The diverse variations of fruit morphology in the Solanaceae family**. (1–3), *Solanum melongena*; (4), *Solanum pimpinellifolium*; (5–8), *Solanum lycopersicum*; (9–14), Variants of *Capsicum annum*; (15), *Physalis alkekengi*; (16), *Physalis floridana*; (17–19), *Physalis philadelphica*. The Chinese lantern in *Physalis* spp. was opened to show the berry inside. Bar = 1 cm.

The Solanaceae family has a rich diversity of fruit types and flower morphologies (Knapp, 2002; He et al., 2004). In addition, this family ranks as one of the most economically important plant families among the angiosperms. Solanaceous fruits represent an important part of the human diet and common fruit crops in this family include tomato (*Solanum lycopersicum*), eggplant (*Solanum melongena*), and chili/pepper (*Capsicum* spp.). The fruits from some species of *Physalis* (such as *Physalis philadelphica* and *Physalis peruviana*) and *Lycium* (e.g., *Lycium barbarum* and *Lycium chinense*) have both curative and culinary usages. Moreover, the Solanaceae family contains many model species for the study of plant developmental processes, including tomato, potato (*Solanum tuberosum*), tobacco (*Nicotiana tabacum*), and *Petunia hybrida*. Thus, this plant family has served as a model for linking genomics and biodiversity (Knapp et al., 2004). The Solanaceous fruits exhibit considerable morphological diversity (Knapp, 2002), including size, shape, and color, both within and between different species (**Figure 1**). How do such morphological variations arise, and what are the underlying genetic bases? In recent years, modern molecular biology and genomic tools have been used extensively to elucidate the mechanisms underlying the evolution and development of these fruit morphological variations. In this review, we summarize the known genetic control of Solanaceous fruit morphological variations, highlight the general mechanisms involved in the evolution of plant morphology, and discuss the future direction.

## Genetic Control of Fruit Size and Shape

Plant fruits exhibit considerable morphological diversity in size and shape. Fruit size and shape variation usually contribute to reproductive isolation of species and have clear evolutionary consequences in natural conditions. Moreover, fruit size is a prime breeding target, and fruit shape is often altered following the size alteration, indicating that the two traits might share a common set of genetic controllers. Solanaceous crops display significant variation in fruit size and shape within and among populations (**Figure 1**). Thus, determination of the genetic basis of these fruit-associated trait variations is the most common type of application-oriented fundamental evolutionary study. Quantitative trait loci (QTLs) for variation of morphological traits between the Solanaceous crops and their closely related wild relatives are well-conserved (Doganlar et al., 2002; Ben Chaim et al., 2003; Frary et al., 2003; Zygier et al., 2005; Borovsky and Paran, 2011; Carvalho et al., 2014; Portis et al., 2014), but most of them have not yet been cloned. Multiple QTLs and/or genes regulating fruit size and shape are well-characterized in the Solanaceae (**Table 1**). The considerable progress in the genetic control of fruit size and shape in tomato was reviewed by van der Knaap et al. (2014). Therefore, in this section we briefly summarize the findings in tomato and focus on the new findings in other Solanaceous species. The identified “fruit morphological variations” QTLs/genes that encode regulators with diverse chemical attributes might form an interaction and regulatory network to control cell division activity/patterns or cell expansion. Therefore, any alteration in these regulators or their pathways may contribute to variations in fruit size and shape.

**Table 1:** QTLs/Genes characterized for variation in fruit size and shape in the Solanaceae.

QTL/gene	Allelic variation	Protein	Process affected	Species	Reference
*fw2.2*	Promoter-Regulatory	A cell number regulator (CNR) family protein	Cell division/fruit size	Tomato eggplant *Physalis*	Frary et al. (2000), Cong et al. (2002), Doganlar et al. (2002), Li and He (2015)
*fw3.2*	Promoter-regulatory	A cytochrome P450 protein	Cell division/fruit size	Tomato pepper	Chakrabarti et al. (2013)
*ovate*	Premature stop	Ovate family proteins	Cell division/fruit shape	Tomato eggplant pepper	Liu et al. (2002), Spinner et al. (2010, 2013), Tsaballa et al. (2011), Drevensek et al. (2012), Gramazio et al. (2014)
*sun*	Transposon insertion-regulatory	A member of the IQD family of calmodulin-binding proteins	Cell division/fruit shape	Tomato eggplant	Xiao et al. (2008), Jiang et al. (2009), Wu et al. (2011), Gramazio et al. (2014)
*fas*	Intron -regulatory	A YABBY-like transcription factor	Cell division/locule number/shape and size	Tomato	Cong et al. (2008)
*lc*	SNPs in downstream-regulatory	A putative ortholog of WUSCHEL	Cell division/locule number/shape and size	Tomato	Munos et al. (2011)
*POS1*	Intron-regulatory	A transcription factors with two CRF-AP2 domains	Cell expansion/fruit size	*Physalis*	Wang et al. (2012, 2014)

### Regulators of Cell Division Activity or Patterns

The two characterized genes that regulate fruit weight are *Fruit weight 2.2* (*FW2.2*) and *Fruit weight 3.2* (*FW3.2*). *FW2.2* is the first cloned QTL in plants (Frary et al., 2000). The allele that increases fruit weight causes an enlargement of the placenta and columella regions of the fruit, which control ~30% fruit size in tomato (Nesbitt and Tanksley, 2001; Cong et al., 2002). A mutation in the *FW2.2* promoter leads to heterochronic expression of the gene during fruit development, resulting in differences in fruit size between cultivated tomato and its wild relatives. FW2.2 is a plasma membrane-anchored protein that is involved in the cell cycle pathway for the control of ovary size (Liu et al., 2003a). *FW3.2* encodes a cytochrome P450 homolog, i.e., the putative ortholog of *Arabidopsis KLUH* (Chakrabarti et al., 2013) and is therefore designated as *S. lycopersicum KLUH* (*SlKLUH*). A mutation located 512 bp upstream of the predicted start of *SlKLUH* transcription is responsible for a change in tomato fruit weight. The increase in fruit weight of *FW3.2* is primarily due to an increase in cell number in the pericarp and septum areas. The putative ortholog of *KLUH* in pepper is also associated with larger fruit suggesting a possible role of the cytochrome P450 family in parallel domestication processes in fruit and vegetable crops (Chakrabarti et al., 2013). The functional conservation of *FW3.2* in angiosperms and the underlying mechanisms require further investigation, whereas the role of FW2.2 in organ size and cell division is highly conserved in most plant species examined. *FW2.2* was found to correspond to a major fruit weight QTL in eggplants (Doganlar et al., 2002). However, *FW2.2* does not play a significant role in controlling fruit size variations between wild and cultivated peppers because pepper fruit has little placental tissue (Zygier et al., 2005). *FW2.2*-like genes have been renamed as the *Cell Number Regulator* (*CNR*) family (Dahan et al., 2010; Guo et al., 2010; Libault et al., 2010; Guo and Simmons, 2011; De Franceschi et al., 2013; Xu et al., 2013; Monforte et al., 2014). CNR family members are localized to the membrane to facilitate the transport of ions (Song et al., 2004; Nakagawa et al., 2007), but the mechanism of regulation of ion transport leading to changes in cell division is unknown. Recently, Li and He (2015) found that *Physalis floridana Cell Number Regulator 1* (*PfCNR1*) encodes a putative ortholog of *FW2.2*. The heterochronic expression levels of *PfCNR1* alleles in ovaries are negatively correlated with cell division activity and berry size variation between different *Physalis* species. PfCNR1 was found to interact with PfAG2, an AGAMOUS (AG) homolog for ovary identity determination (Yanofsky et al., 1990). Moreover, PfAG2 binds to the CArG-box in the *PfCYCD2;1* promoter to repress the expression of this gene. The work in *Physalis* suggests a novel mechanism mediated by an MADS-domain protein for a cell membrane-localized protein to control cell division suggesting a molecular link between ovary identity and growth in plants (Li and He, 2015).

Fruit elongation is an important feature that affects fruit shape. Elongation in the tomato fruit is controlled mainly by *OVATE* and *SUN*. *OVATE* encodes a member of the ovate family proteins (OFPs), and a mutation that results in a premature stop codon leads to the pear-shaped fruit in tomato (Liu et al., 2002). The *Arabidopsis* OFP members act as transcriptional repressors in controlling cell elongation, plant growth, and development (Wang et al., 2007, 2011). Yeast two-hybrid screens using the tomato OVATE as bait identified the TONNEAU1-recruiting motif (TRM) superfamily as prey. TONNEAUs (TON) and TON-TRM interaction play critical roles in preprophase band formation and microtubule array organization of plant cell division and cell elongation (Spinner et al., 2010, 2013; Drevensek et al., 2012). Thus, an interaction between OVATE and TRMs may provide a mechanistic link between fruit patterning and growth, nonetheless, this assumption needs substantiation. OFPs are present in all major lineages of land plants (Liu et al., 2014); whether they shared a conserved role needs to be investigated. At least *Ovate*-like genes from pepper and eggplants are also involved in determining fruit shape (Tsaballa et al., 2011; Gramazio et al., 2014). *SUN* encodes a member of calmodulin-binding proteins (Xiao et al., 2008, 2009) and regulates vegetative growth and reproductive organ shape by changing cell division patterns (Wu et al., 2011). Wild-type *SUN* is only expressed 10 days post-anthesis fruit (van der Knaap et al., 2014); however, a transposition of unusual 24.7 kb duplication event mediated by the retrotransposon *Rider* causes mutations in some tomato cultivars (Jiang et al., 2009). This leads to greater expression in the entire floral and fruit development, and elongated fruit (Xiao et al., 2008, 2009). How *SUN* regulates cell division pattern remains unclear, but *SUN* ortholog also controls the fruit shape in eggplant (Gramazio et al., 2014) implicating a conserved developmental role of this gene family.

Alteration in the locule number frequently affects both fruit shape and size. For example, the wild species *Solanum pimpinellifolium* commonly contain two to four locules while tomato cultivars have more; and in extreme cases, more than eight locules have been observed (Munos et al., 2011). Most phenotypic variation due to locule number variation is explained by *fasciated* (*fas*) and *locule number* (*lc*). *FAS* encodes a *YABBY-*like transcription factor *SlYABBY2* (Cong et al., 2008). The mutation *fas*, which resulted from a 294-kb inversion with one of the breakpoints in the first intron of *SlYABBY2*, led to the increases in locule number, and was a critical step in the production of extreme fruit size during tomato domestication (Cong et al., 2008). However, the details of how *SlYABBY2* impacts locule number in tomato are not well-understood. *LC* was identified to be associated with two single nucleotide polymorphisms (SNPs) located 1080 bp downstream of the putative tomato ortholog of *WUSCHEL* (*WUS*), a homeodomain transcription factor (Clark, 2001; Munos et al., 2011). *LC* controls the number of carpel primordia and a mutation results in a fruit with more than the typical two to three locules. Since increased expression of *AtWUS* leads to increased floral organ number in *Arabidopsis* (Clark, 2001), *SlWUS* is the most likely candidate to underlie *lc*. *AtWUS* positively regulates *AtAG* while *AtAG* down-regulates *AtWUS*; the down-regulation is mediated by two downstream CArG *cis*-regulatory elements bound by AtAG (Lenhard et al., 2001; Lohmann et al., 2001; Liu et al., 2011). In tomato, the two SNPs associated with *lc* are located in a putative CArG *cis*-regulatory element, but surprisingly, a considerable change in the expression of *SlWUS* compared with the wild type was not observed (Munos et al., 2011). More evidence is needed to verify the role of *SlWUS* in the control of fruit size, or to link *lc* and *SlWUS*.

### Cell Expansion Regulators

The above-characterized genetic regulators mainly affect cell division activity or patterns; however, alteration in cell expansion also plays a role in the evolution of fruit size. Pericarp size, particularly pericarp thickness, is a strong determinant of *Solanaceous* fruit size. Pericarp thickness appears to be governed by endoreduplication (Cheniclet et al., 2005). Endoreduplication – arrest in mitotic activity accompanied by a concomitant increase in nuclear DNA levels during fruit development – is believed to drive cell expansion, and is mainly regulated by cell cycle genes (Chevalier et al., 2011). During tomato fruit development, endoreduplication acts as an important morphogenetic factor supporting cell growth and multiple physiological functions (Chevalier et al., 2014). Impairment in the expression of *WEE1*, which encodes the cell cycle-associated protein kinase in transgenic tomato plants, results in a reduction in plant and fruit size, because of decrease in cell size that correlates with a decrease in the DNA ploidy levels (Gonzalez et al., 2007). Downregulation of tomato *CELL CYCLE SWITCH A 52 kDa* (*SlCCS52A*) does not affect the number of pericarp cell layers, but results in the formation of significantly smaller fruit, along with a sharp reduction in the ploidy level and pericarp cell size (Mathieu-Rivet et al., 2010a,b). The auxin *Sl-IAA17* transcriptional repressor also controls tomato fruit size by regulating endoreduplication-related cell expansion (Su et al., 2014). The role of endoreduplication in increased cell expansion in fruit development is controversial (Chevalier et al., 2011; Nafati et al., 2011), and its role in natural variation of fruit size is not known. Recently, a key cell expansion regulator was characterized in *P. philadelphica* (tomatillo). The characterized gene is *Physalis Organ Size 1* (*POS1*), previously designated as *Pp30*, which encodes a putative transcription factor with two CRF (cytokinin response factor)-AP2 (APETALA2) domains, and positively controls floral and fruit organ sizes in tomatillos (Wang et al., 2012, 2014). The expression levels of the *POS1* gene were positively associated with size variation in tomatillo reproductive organs such flowers, berries and seeds. *POS1* knockdown resulted in smaller flowers and berries with smaller cells compared with their wild type counterparts. Conversely, *POS1* overexpression promoted organ size without increasing the cell number. The first introns of the *POS1* alleles from large, intermediate and small tomatillo groups contained one, two and three 37-bp repeats, respectively. Furthermore, copy variation of repeats in the first intron of *POS1* alleles resulted in differential expression of this gene. Thus, the novel regulatory variation in *POS1* regulates reproductive organ size variation in tomatillos (Wang et al., 2014).

## Genetic Basis of Fruit Color Variation

Fruit color is essential for attracting animals and humans, and thus, facilitates seed dispersal. Color is determined by different proportion of surface pigments, such as carotenoids, chlorophyll, flavonoids, and anthocyanins (Liu et al., 2003b; Nashilevitz et al., 2010; Kachanovsky et al., 2012). The color of berries varies widely and can be red, purple, orange, yellow, or green (**Figure 1**). Brightly colored berries generally tend to be juicy and extremely soft, whereas, mature green berries are harder and have a woody texture (Symon, 1987). Phylogenetic reconstructions suggest that green fruits belong to the primitive clade, whereas brightly colored (red, orange, yellow) species are derived clades (Peralta and Spooner, 2001). Several genes were characterized in tomato, pepper, and eggplant. Carotenoid content is the primary determinant of fruit color that affects nutritional value and appearance. In the carotenoid pathway, color diversity depends on the quantity of pigment produced, and the point where the pathway is arrested. Many transcription factors participate in controlling this pathway. Rodriguez-Uribe et al. (2012) determined the carotenoid composition in a number of orange-colored pepper fruit, and compared it with transcript abundance for the carotenogenic enzymes, such as phytoene synthase (Psy), lycopene β-cyclase (LcyB), β-carotene hydroxylase (CrtZ), and capsanthin-capsorubin synthase (Ccs). A splicing mutation in the *Psy* gene *1* (*Psy1*) causes orange coloration in Habanero pepper fruits (Kim et al., 2010). A chimeric transcript containing *Psy1* and a potential mRNA is associated with *yellow flesh* color in tomato accession PI114490 (Kang et al., 2014). The single dominant tomato *LcyB* gene increases β-carotene in the fruit while *old-gold (og)*, a recessive mutation of the *LcyB* abolishes β-carotene and increases lycopene (Ronen et al., 2000). LcyB is homologous to Ccs, an enzyme that produces red xanthophylls in pepper fruits (Ronen et al., 2000). A tandem repeat structure in the promoter region of *Ccs* causes the yellow fruit color in pepper (Li et al., 2013). *CrtZ* mutant results in accumulation of β-carotene and conversion of red to orange color in pepper fruit (Borovsky et al., 2013). Nowadays, many QTLs/genes were found to be involved in fruit coloration through affecting plastid characteristics. QTL *pc8.1*, affects carotenoid content in pepper fruit and is associated with variation in plastid compartment size (Brand et al., 2012). The variations in chromoplasts are associated with carotenoid compositional differences and fruit color of different pepper cultivars (Kilcrease et al., 2013). An *Arabidopsis pseudo response regulator 2-like* (*APRR2-like*) gene is linked to pigment accumulation in tomato and pepper fruits. Overexpressing this gene in tomato increased plastid number, area, and pigment content; thus, enhancing the levels of chlorophyll in immature unripe fruits and carotenoids in red ripe fruits (Pan et al., 2013). The positions of ten genes in the carotenoid biosynthetic pathway of pepper were homologous with the positions of the same genes in tomato (Thorup et al., 2000). Amino acid substitutions in homologs of the STAY-GREEN protein of rice are responsible for green-flesh and chlorophyll retainer mutations of tomato and pepper (Barry et al., 2008). *CaGLK2*, a GOLDEN2-like transcription factor regulates natural variation of chlorophyll content and fruit color in pepper fruit (Brand et al., 2014). Virus-induced gene silencing (VIGS) of *SlMYB12* resulted in a decrease in the accumulation of naringenin chalcone, and pink-colored tomato fruit, suggesting an important role for this gene in regulating the flavonoid pathway in tomato fruit (Ballester et al., 2010). Major anthocyanin in eggplant peel was delphinidin-3-rutinoside while the predominant pigment in violet pepper was delphinidin-3-*trans*-coumaroylrutinoside-5-glucoside (Sadilova et al., 2006). It is possible that a conserved complex regulatory pathway controls Solanaceous fruit colors, but the complete genetic components in the carotenoid regulatory pathway have not yet been revealed, even in a Solanaceous species.

## The Evolution of the Fruit Morphological Novelty

**FIGURE 2:**
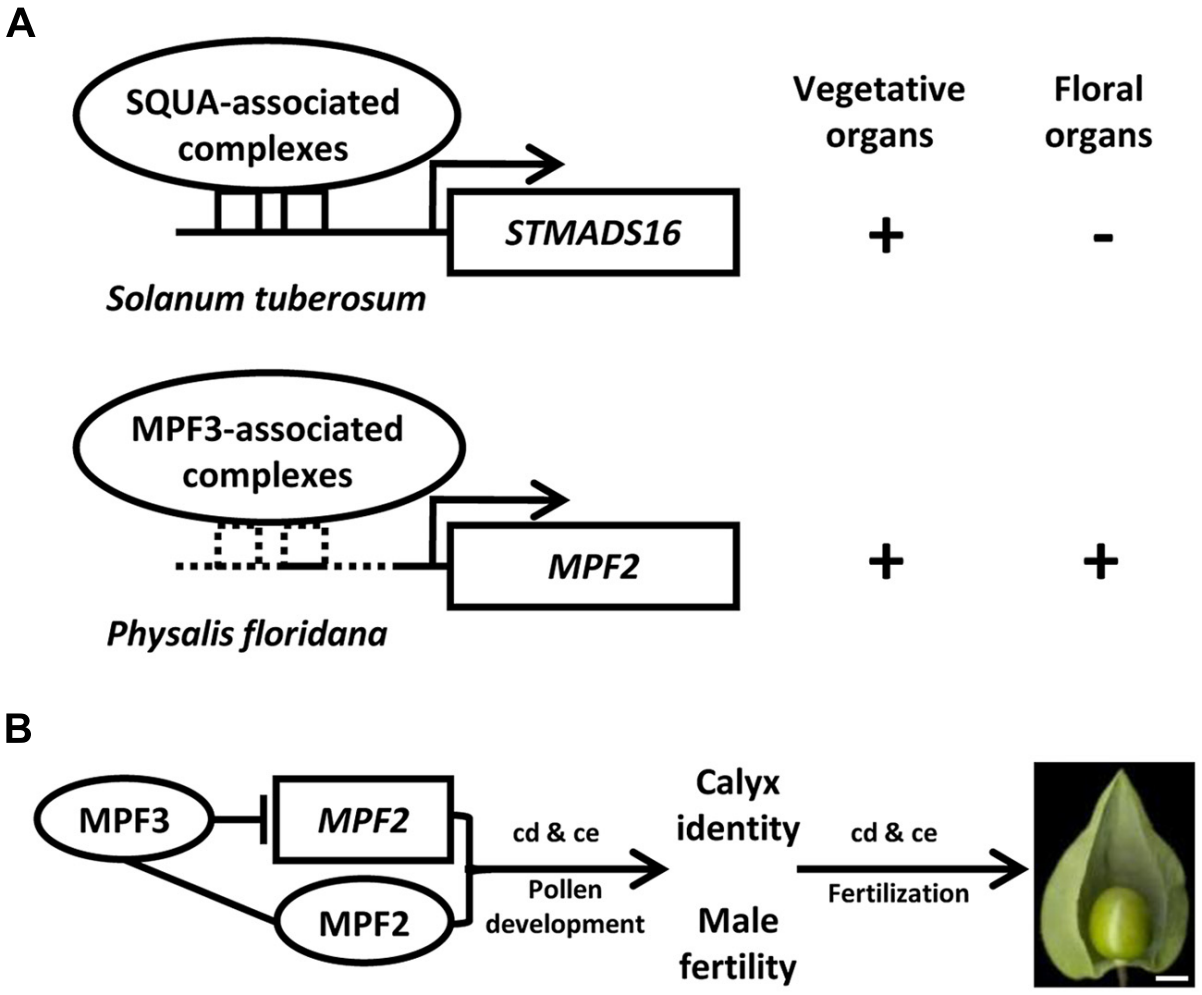
**Genetic bases underlying the origin of the morphological novelty the Chinese lantern in *Physalis*. (A)** Schematic diagram of the heterotopic expression of *MPF2* in floral organs. *MPF3* is the putative ortholog of *SQUAMOSA* (*SQUA*). In *S. tuberosum*, SQUA-like protein associated complex binds to the CArG-boxes in *STMADS16* promoter and represses its expression in floral organs. In *P. floridana*, the sequence alteration in the *MPF2* promoter provides a possibility to loosen the repression of MPF3-associated complex, thus leading to the heterotopic expression of *MPF2* in floral organs. Arrows indicate the transcription initiation sites. Sequence divergence in *STMADS16* and *MPF2* promoters is depicted by the solid and the dashed lines. The solid square boxes indicate CArG-boxes in the *STMADS16* promoter, and the dashed square boxes indicate altered CArG-boxes in the *MPF2* promoter. The plus or minus represents the gene expression or not. **(B)** The MPF3 -*MPF2*/MPF2 interactions involve in the development of the Chinese lantern in *Physalis*. The line indicates the interaction of the two components. The blocked line stands for repression of gene expression. Arrows represent regulations. cd, cell division; ce, cell expansion. Bar = 5 mm. In both **(A,B)**, proteins are presented in Roman and ovals, and genes are presented in italics and rectangles.

The origin of morphological novelties is a long-standing dispute in evolutionary biology. An understanding of this process demands the elucidation of the developmental and genetic mechanisms that produce such structures. Unlike *Solanum* and *Capsicum* species, *Physalis* has distinguished fruit morphology with a papery husk as the accessory trait of fruits (Whitson and Manos, 2005). The distinct trait of *Physalis* species is termed the Chinese lantern or the inflated calyx syndrome (ICS) since it is a derivative of the calyx (He and Saedler, 2005). Within the Solanaceae, only five genera (*Physalis*, *Withania, Przewalskia, Margaranthus*, and *Nicandra*) feature this morphological novelty. The nature of the Chinese lantern is an inflated fruiting calyx, and fertilization/hormonal signals trigger the formation of ICS in *Physalis* and *Withania* (He and Saedler, 2005, 2007; Khan et al., 2012). A series of microevolutionary time scale studies revealed that the origin of the Chinese lantern is associated with the heterotopic expression of the *Physalis* MADS-box gene 2 (*MPF2*) in floral organs. Moreover, its identity is determined by another *Physalis* MADS-box gene 3 (*MPF3*; He and Saedler, 2005; Zhao et al., 2013). Unlike its ortholog of *S. tuberosum* MADS-box gene 16 (*STMADS16*), *MPF2* is heterotopically expressed in the floral organs of *Physalis* (**Figure 2A**). The heterotopic expression of *MPF2* may result from the variation in the CArG-boxes in its promoter. The phenotypic variation of *MPF2* knockdowns further supports the role of MPF2 in male fertility and fruiting calyx growth (He and Saedler, 2005). Thus, heterotopic expression of *MPF2* is the key to the origin of the Chinese lantern morphology. While *MPF3* is specifically expressed in floral tissues, this gene encodes a euAP1 MADS-domain protein, which is primarily localized to the nucleus, and it interacts with MPF2 and some floral MADS-domain proteins to selectively bind the variants of CArG-boxes in the *MPF2* promoter (**Figure 2A**, Zhao et al., 2013). Besides the role in calyx identity, MPF3 regulates ICS formation and male fertility through interactions with the *MPF2* gene or MPF2 protein (**Figure 2B**, Zhao et al., 2013). The ICS-determined genes also function in male fertility; either in pollen maturation or yields, and their encoding proteins also interact with floral MADS-proteins for stamen development (He et al., 2007). *MPF3* downregulation increase *MPF2* expression significantly in the calyces and androecium; however, the expression of *MPF3* is not affected in *MPF2*-downregulated flowers (Zhao et al., 2013). Therefore, the novel role of the MPF3-*MPF2* regulatory circuit in male fertility is integral to the origin of the Chinese lantern. Thus, any molecular interactions associated with MPF2 and MPF3 may contribute to ICS formation (He et al., 2007; Zhao et al., 2013). Dissecting the *double-layered-lantern mutant1* (*doll1*), a *P. floridana GLOBOSA* (*GLO*)-like MADS-box gene 1 (*PFGLO1*) genomic locus deletion mutant (Zhang et al., 2014a) further suggested a role of male fertility in the development of the Chinese lantern in *Physalis*. The corolla and androecium of *doll1* are respectively transformed into the calyces and gynoecium (Zhang et al., 2014a). On the other hand, downregulating *PFGLO2*, the paralog of *PFGLO1* impaired male fertility (Zhang et al., 2015). Further evolutionary analyses suggest that the evolution of ICS in Solanaceae is associated mainly with divergence related to *MPF2*-like genes, and alteration in *MPF2*-related molecular traits plays a crucial role (Hu and Saedler, 2007; Khan et al., 2009; Zhang et al., 2012).

## Future Research Highlights

### The Evolution of Fruit Type in Solanaceae

**FIGURE 3:**
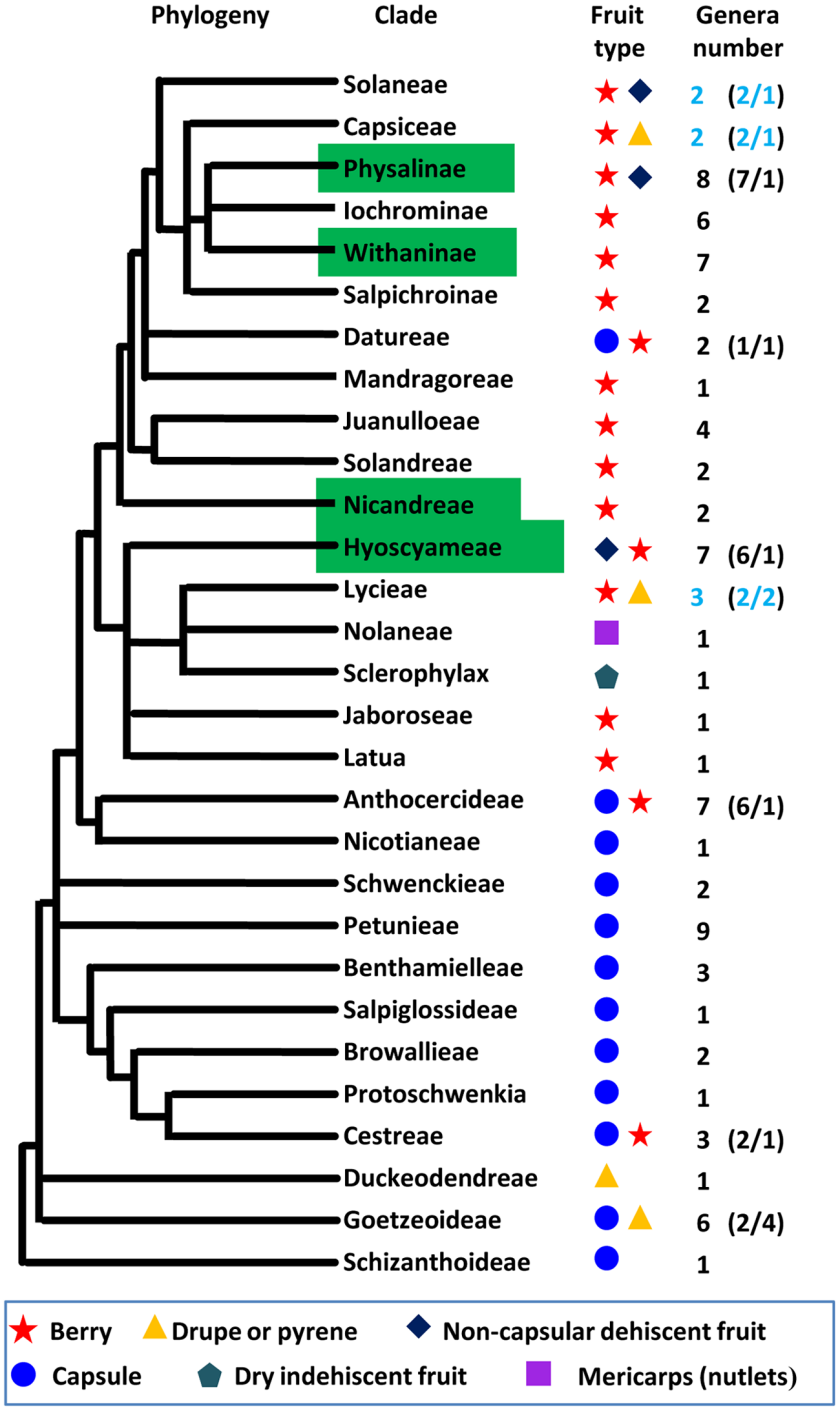
**The evolution of fruit types within the Solanaceae**. The topology of Solanaceous phylogeny was deduced from the molecular phylogeny trees using the combined *ndhF* and *trnLF* sequences (Olmstead et al., 2008). The definition of fruit type was adopted from the previous work (Knapp, 2002; Olmstead et al., 2008). The blue circle, yellow triangle, purple square, black rhombus, red star, and aqua Pentagon represent fruit type as indicated. The clade features ICS was indicated with a green box. The number of genera in each clade and the ration of genera with different fruit type in some clades (in parenthesis) are given, and coexistence of two fruit types in one genus is marked in blue.

Fruit is the vehicle for seed dispersal, and the origin of the fruit is an evolutionary adaptation that facilitates survival and distribution of progeny. Thus, the evolution of fruit morphology is under strong selective pressures. Fruit size and shape are mostly related to domesticated crops; however, fruit type is a key adaptive feature to terrestrial habitats in natural conditions. Therefore, revealing the genetic basis of the alteration of fruit types should be a theme of future evolutionary research. Berry (fleshy fruit), capsule, drupe, dry indehiscent fruit, non-capsular dehiscent fruit, and mericarp are the six known types of Solanaceous fruits (Knapp, 2002; Olmstead et al., 2008). We mapped the six fruit types on the phylogenetic tree of the family Solanaceae (**Figure 3**). Berry and capsule are apparently predominant types of fruits. Capsules occur in the most basal clades and broadly distribute in basal taxa while the origin of berry happened in Cestreae but became predominant after the origin of Anthocercideae. Berry covered by ICS seems to have multiple independent origins in Physalinae, Withaninae, Nicandreae, and Hyoscyameae. Non-capsular dehiscent fruit occurs independently in Solanaceae, Physalinae and Hyoscyameae. Drupe and pyrene occur at least twice in Duckeodendreae, Goetzeoideae, and Lycieae. Dry indehiscent fruit is only observed in Sclerophylax. The genetic control of fruit type is not well-studied, and as the research is hampered by large evolutionary and genetic distance among plants with different fruit types. However, the evolutionary genetic control regarding the transition between the various fruit types will be an extremely interesting target. Thus, coexistence of two fruit types in some Solanaceous clades, particularly in a genus from each clade of Solanaceae, Capsiceae, and Lycieae (**Figure 3**) provides a good system for comparison to understand the genetic variation causing such morphological divergence.

### Natural Variation and Domestication

Natural variation of a trait, even the maintenance of a morphological novelty, is a consequence of adaptation to natural environments. The Solanaceae family displays considerable diversities at different levels and is therefore proposed as a good model to study the evolutionary mechanisms of biodiversity (Knapp et al., 2004). Several model plant species have been established in laboratory experiments, such as tomato, and this will facilitate the work in this family. However, diversity and natural variation are poorly evaluated within the phylogenetic context, and, therefore, the evolutionary mechanisms are not well-understood. In particular, the origin of morphological novelties and the evolution of fruit type (**Figure 3**) have long been overlooked. Besides the Chinese lantern, other novel morphological traits need to be identified. In addition, the Solanaceae family contains fleshy fruits and vegetables such as tomato, eggplant, chili/pepper, and tomatillo that are eaten by humans, thus several species are domesticated crops and are bred for their diverse morphologies (**Figure 1**). How human selection affects the genomes of these species, compared with their closely related wild relatives and plants with other fruit types, and further creates ideal traits to meet people’s demands is not well-known. Understanding the processes of how plants respond to alteration of natural and/or human environments are the most fundamental to understand the process of life and should, therefore, be a high priority for research.

### Enhancing the Work in the New Model *Physalis*

The fruit morphology in *Physalis* varies significantly (**Figure 1**). This genus displays the post-floral morphological novelty Chinese lantern, and the color, flavor, and size of the berries show an impressive variability. A few *Physalis* species, such as *P. peruviana*, *P. philadelphica*, *P. alkekengi*, and *P. angulata* are becoming new leading Solanaceous horticultural and medicinal crops. With efforts in our group in recent years, evolutionary developmental genetics of *Physalis* fruits (berry and ICS) are now understood. In particular, many molecular research tools have been established in *Physalis*, including gene isolation, gene expression detection, protein-protein interactions, transformation system, and VIGS approach (He and Saedler, 2005; He et al., 2007; Zhao et al., 2013; Wang et al., 2014; Zhang et al., 2014a,b; Li and He, 2015). In this respect, *Physalis* has, therefore, has been established as an emerging model plant for development, evolution and ecology. The genetic repertoire for berry and ICS development via genetic and genomic tools needs further investigation. Moreover, multiple experimental approaches will help understand the selective values of the Chinese lantern, and the evolutionary mechanisms of variations in berry size, color, and medicinal components of *Physalis*.

Instead of domesticated crops, more wild plants have to be included. Comparative analyses of Solanaceous crops and their wild relatives will bring new insights into growth, development, and evolution. Thus, comparative microevolutionary-scale studies between closely related genera/species at different levels, including the development, the cellular process, and genetic variation in a phylogenetic context, are major themes in evolutionary developmental genetics of fruit morphological variation.

## Conclusion

The evolution of morphological variation is a consequence of adaptive evolution. Advances in genetics and genomics provide genetic and molecular tools that have facilitated the map-based and candidate-gene-based cloning of several key genes in fruit development, creating new inroads into understanding the primary regulatory mechanisms underlying fruit morphological variation. Recruitment of a preexisting (regulatory) gene frequently occurs. The recruiting mechanisms include alteration of gene expression and/or gene function through mutations in the regulatory and/or coding regions. The regulatory motifs are often demonstrated to be located in the promoter or the intron, and altering them may cause heterotopic (alteration of expression place), heterochronic (change of expression time), ectopic (a high expression level), or downregulated expression of a gene that appears to play a predominant role in the evolution of plant morphology. Species-specific evolution cannot be excluded; however, independently recruiting the same genetic variation, and regulatory networks could to some extent explain the multiple origins of a particular trait state. In addition, multiple losses of a trait state may occur because once the interacting and regulatory networks have been established for a trait, the evolutionary pattern of that trait may be determined by any secondary mutation in the trait biosynthetic pathways.

In the coming years, there will be considerable focus on isolating new developmental genes and bridging the gap between these genes and their functions. Understanding their recruiting mechanisms and interactions with environments to determine fruit morphological variation in a phylogenetic context are key scientific questions in evolutionary developmental biology. Translation of the information from a few model plants to the large portion of remaining non-model species should be improved. These results will provide fundamental insights into plant developmental processes as well as help to establish novel strategies to improve the productivity and fruit quality of crops.

## Author Contributions

CH designed the work; LW constructed **Figure 1** and **Table 1**; JZ drew **Figure 2**; JL and LW compiled the **Figure 3**; WL and CH wrote the manuscript; JZ and JL contributed critically to the editing the manuscript, and approved the manuscript.

## Conflict of Interest Statement

The authors declare that the research was conducted in the absence of any commercial or financial relationships that could be construed as a potential conflict of interest.
